# Phytomedicine in Otorhinolaryngology and Pulmonology: Clinical Trials with Herbal Remedies

**DOI:** 10.3390/ph5080853

**Published:** 2012-08-20

**Authors:** Koosha Ghazi Moghadam, Hasan Mete Inançlı, Nazanin Bazazy, Peter K. Plinkert, Thomas Efferth, Serkan Sertel

**Affiliations:** 1 Department of Otorhinolaryngology, Head & Neck Surgery, University of Heidelberg, Im Neuenheimer Feld 400, 69120 Heidelberg, Germany; 2 Molecular Mechanisms of Head and Neck T—A102, Division Signal Transduction and Growth Control, German Cancer Research Center (DKFZ), Im Neuenheimer Feld 280, 69120 Heidelberg, Germany; 3 Department of Otorhinolaryngology, Near East University Hospital, 922022 Lefkosa, Turkish Republic of Northern Cyprus; 4 Department of Pharmaceutical Biology, Institute of Pharmacy and Biochemistry, University of Mainz, Staudingerweg 5, 55099 Mainz, Germany

**Keywords:** phytomedicine, herbal remedy, clinical trial, evidence-based, otorhinolaryngology, pulmonology

## Abstract

Phytomedicine has become an important alternative treatment option for patients in the Western world, as they seek to be treated in a holistic and natural way after an unsatisfactory response to conventional drugs. Ever since herbal remedies have been introduced in the Western world, clinicians have raised concerns over their efficacy and possible side-effects. A PubMed (Medline) search was performed covering the last five years (01/07–04/12) and including 55 prospective clinical randomized control trials in the medical specialities Otorhinolaryngology and Pulmonology. In this review, we present evidence-based clinical data with herbal remedies and try to enlighten the question of efficacy and reliability of phytomedicine.

## 1. Introduction

Phytomedicine is defined as the use of plants, parts of plants as well as isolated phytochemicals for the prevention and treatment of various health concerns [1]. Medical treatments cover the application of different components of plants (blossom, leaf, stem, radix), aromatic essential oils and herbal extracts as herbal teas, via massage as packs or wraps or in therapies using water, steam or inhalation [2]. Phytomedicine is covered in many traditional medicines, e.g., Traditional Chinese Medicine (TCM) or Kampo Medicine [2,3,4]. Herbal remedies have played an important role in treatment from ancient to modern times. Although these subjects lost their importance in 20th century because of the modern synthetic treatments, there is a renewed interest today in medicinal plants usage as natural products for the generation of semi-synthetic derivatives [2].

Phytomedicine has become an important alternative treatment option for patients in the Western world, as they seek to be treated in a holistic and natural way after an unsatisfactory response to conventional drugs. In fact, patients living in Eastern countries with a long history of traditional medicines regularly use herbal drug prescriptions, as Western medications are hardly affordable for the majority of the population. Ever since herbal remedies have been introduced in the Western world, clinicians have always raised concerns over their efficacy and possible side-effects. 

The application of phytomedicines, especially in private practices, is booming. In Western Europe annual revenues reached US$ 5 billion in 2003–2004, while in China sales of products totaled US$ 14 billion in 2005 [5]. This development is founded in patients’ desire to be treated in a holistic and natural way. Moreover, phytomedicines provide valuable resources to meet the requirements for global health care at affordable prices [6]. According to the World Health Organization (WHO), nearly 80% of the population of developing countries, even nowadays, relies on phytomedicines as their only affordable source of medication [7]. The question arises whether this development is justified and can be objectively proven by controlled, randomized clinical trials. Recognition and application of phytomedicine into Western medicine depends most importantly on evidence-based clinical data. In this review, we focus on evidence-based clinical data with herbal remedies in the treatment of diseases in Otorhinolaryngology and Pulmonology.

## 2. Material and Methods

A PubMed (Medline) search was performed on international publications covering the last five years from January 2007 until April 2012, complying following inclusion and exclusion criteria. The search was limited to the terms “phytomedicine”, “herbal drug”, “herbal medicine”, “natural product”, “natural drug”, “traditional medicine” and frequently used medicinal plants. Inclusion criteria in selecting the publications were set as following: Type of article: clinical trials and randomized controlled trials; Species: humans; Language: English. In addition, clinical trials were selected as to medical branch, scientific quality of study design and clinical impact. As to medical branches, the focus was set on Otorhinolaryngology and Pulmonology. Only prospective, randomized, placebo-controlled, and double-blind designed trials were selected as golden standard of clinical trials.

Exclusion criteria in selecting the publications were phase I studies, case series, and retrospective studies. Supplementary, we performed a proactive Medline search on frequently used medicinal plants 

(*Ginseng*, *Echinacea*, *Salvia*, and *Ginkgo biloba*) in the medical branches mentioned above, which we did not found in our initial search and included interesting publications in this review. In addition, we performed a Medline Search with “phytotherapy” as a Medical Subjects Heading (MeSH) term with the mentioned limits and subsequently checked the references available for each article.

## 3. Results

### 3.1. Rhinitis and Sinusitis

Allergic rhinitis is associated with sneezing, nasal discharge, and nasal obstruction. The common therapy strategy of allergic rhinits is usually on avoidance or removal of antigens and symptom therapy with antihistamines and topical steroids, but the potential side-effects of these drugs with long-term usage are of concern. Patients have demanded alternative therapy options, especially with traditional and herbal medicine. In Table 1 various herbal remedies in the treatment of allergic rhinitis and acute rhinosinusitis are listed.

*Naringenin chalcone *as the effective constituent of tomato extracts has been shown to inhibit the release of histamine from mast cells in the initial phase of inflammation and decrease the eosinophils and eosinophil cationic proteins. In a randomized double blind study from Japan, the effects of tomato( *Lycopersicon esculentum *Miller) extracts (TE, 360 mg per day for eight weeks) on patients with mild to moderate perennial allergic rhinitis were studied. In the TE group, nasal obstruction, rhinorrhea, and sneezing were improved significantly compared to placebo (*p* < 0.05) [8]. 

In another study, a combined herbal therapy (ARND) against perennial allergic rhinitis was tested in a randomized, double-blinded and placebo-controlled setting, with a crossover arrangement for the administration of allergic rhinitis nasal drop or placebo. ARND consisted of *Herba centipedae *23%, *Herba menthae* 16%, *Radix paeoniae alba* 16%, *Radix scutellariae* 10%, *Radix glycyrrhizae* 6%, *Radix platcodi* 6%, *Flos lonicerae* 5%, *Fructus zizyphi jujubae* 5%, *Rhizoma coptidis* 4%, *Radix ledebouriellae* 5%, and *Pericarpium citri reticulatae* 4%. Each of these herbal remedies has anti-bacterial and anti-inflammatory activity. ARND relieved clinical symptoms in patients with perennial allergic rhinitis and improved their quality of life [9]. 

Effects of another combined herbal therapy against allergic rhinitis were studied in a randomized, double-blinded crossover trial. The herbal tablet consisting of *Cinnamomum zeylanicum, Malphighia glabra* and *Bidens pilosa *were compared to the positive control treatment with loratadine and placebo. The herbal tablet significantly reduced nasal symptoms and inhibited the release of prostaglandin D2 in nasal lavage analysis [10].

*Shi-Bi-Lin *(SBL) a herbal remedy consisting of 7.5 g *Xanthium sibiricum *Patrin ex Widder* (Fructus asteraceae)*, 20 g *Angelica dahurica *(Fisch. ex Hoffm.) Benth*. (Radix apiaceae)*, 7.5 g *Saposhnikovia divaricata *(Turcz.) Schischk*. (Radix apiaceae)*, 15 g *Magnolia biondii *Pamp. *(Flos magnoliae)*, 5 g* Gentiana scabra *Bunge* (Radix gentianaceae)* and 5 g *Verbena officinalis *L. *(Herba verbenaceae) *has been shown to suppress eosinophil infiltration in animals’ nasal tissues and inhibited the expression of endothelial nitric oxide synthase and release of thromboxane B2, which are playing an important role in the acute phase of inflammation [11]. In a randomized, double-blinded, placebo-controlled study, there were no significant improvements in the symptoms of patients with perennial allergic rhinitis treated with SBL in comparison to placebo as control for a limited period of 4 weeks [12].

Dust mite allergy is one of the most common reasons for allergic rhinitis. *Ten-cha (Rubus suavissimus) *represents a common phytotherapeutic against allergic rhinitis in Japan. In a randomized, double-blinded, placebo-controlled study *ten-cha* (with dosage of 400 mg three times per day) could improve nasal symptoms without showing significant differences to the placebo [13]. In another study from Croatia, Matkovic *et al. *investigated the effect of *Astragalus membranaceus *(AM)*,* a herbal remedy with immunomodulatory characteristics. In their double-blinded, placebo-controlled clinical trial AM significantly decreased the intensity of rhinorrhea without any significant changes in IgE level and eosinophil counts in patients in comparison to placebo [14].

*Nigella sativa*, a plant mostly used in traditional medicine in the Middle East and Mediterranean area, has been described with anti-inflammatory and anti-allergic effects through changes in activity of phagocytosis and killing activity of polymorphonuclear (PMN) leucocytes, leukotriene synthesis, and inhibition of histamine release [15]. Its effect on patients with allergic rhinitis has been investigated in two studies. *Nigella sativa *seeds have been shown to significantly increase phagocytosis and killing activity of PMNs and count of T-cell receptors in patients after immunotherapy rather than patients receiving conventional immunotherapy [16]. In another study, patients treated with *Nigella sativa *oil for 30 days had significant improvements of nasal signs and symptoms [17].

Idiopathic rhinitis (IR), as one differential diagnosis to perennial rhinitis, is an impairment of nasal breathing accompanied by sneezing, nasal itching, and rhinorrhoea. Dysregulation in the neurogenic mechanism of blood supply to the cavernous tissue of the inferior turbinate is discussed as the major pathomechanism of IR [18,19]. Neuropeptides like substance P from the sensory neurons of nasal mucosa are claimed to play a major role in the neurogenic mechanism of IR. Capsaicin, the active component of plants belonging to the genus *Capsicum*, interacts with sensory neurons [20]. Intranasal application of *Capsicum* spray in IR was evaluated in a randomized placebo-controlled trial. *Capsicum *(4 mg/puff, three times a day for three consecutive days) showed a significant reduction in the frequency of IR symptoms compared to controls [21].

Acute rhinosinusitis is one of the most frequently treated diseases in Otorhinolaryngology. A root extract of *Pelargonium sidoides*, originating from South Africa and traditionally used as a medicinal plant by the Zulu, is used in Europe against acute bronchitis, tonsillopharyngitis, and common cold [22,23]. In a randomized, double-blinded, placebo-controlled trial *Pelargonium sidoides* (Umckaloabo) extract EPs^®^ 7630 influenced the course of acute rhinosinusitis favourably and caused a faster recovery compared to placebo [24]. *Pelargonium sidoides* has been described as an immune stimulant, inhibitor of bacterial adhesion to mucus and also multiplier of cilliary beat frequency [25]. In a randomized, double-blinded study, treatment with a dosage of 30 drops three times a day with *Pelargonium sidoides*, yielded an improvement of sinus severity scores after seven days [24]. However, Umckaloabo is suspected of being hepatotoxic [26]. Eucalyptus oil is one of the most famous phytomedicines used by patients and physicians against acute rhinosinusitis. The main constituent of eucalyptus oil is cineole. Its efficacy and safety against acute rhinosinusitis were proven in a placebo-controlled, double-blinded and randomized clinical trial [27]. Remarkably, treatment of purulent rhinosinusitis with cineole reduced symptoms more effectively and statistically significant compared to an alternative herbal preparation composed of five different medicinal plants (12 mg *Gentianae radix*, 36 mg *Primulae flos cum calycibus*, 36 mg *Ramicis herba*, 36 mg *Sambuci flos*, and 36 mg *Verbenae herba*) [28]. In this case, cineole as the main constituent of eucalyptus is seen clinically as superior to a combination of medicinal herbs, which are supposed to be effective against acute rhinosinusitis. This represents an exception as therapeutic efficacy of phytomedicine is based on synergistic or antagonistic interaction of many phytochemicals [7].

### 3.2. Olfactory Loss

Olfactory loss happens to be a complication after acute and chronic sinusitis due to viral infections. Swelling of the olfactory cleft after infection and degeneration of neurosensoral function are mentioned as its pathgenesis. *Ginkgo biloba *has been attributed to olfactory regeneration in animal models. In a well-designed, double-blinded, controlled trial a combination therapy of *Ginkgo biloba* with mometasone nasal spray was compared to mometasone monotherapy in patients with postviral olfactory loss. The results show significant improvements of odor threshold and identification in the combination therapy group [29].

### 3.3. Asthma

Asthma is defined as a recurrent and reversible obstruction of the respiratory tract with bronchospasm, cough, wheezing, and dyspnea. Avoidance of allergens and the application of inhalative sympathomimetics, oral or inhalative corticosteroids, and leukotriene antagonists are the main therapeutic lines. Here, we focus on phytomedicines with clinical evidences in the treatment of asthma (Table 2).

*Nigella sativa* has been found as a histamine antagonist and inhibitor of the histamine receptor. It is certified as anti-inflammatory, anti-tussive and anti-cholinergic as well. A placebo-controlled trial has shown that treatment of asthmatic patients with an extract of *Nigella sativa* for three months improved symptoms and pulmonary function tests [30]. French maritime pine bark is well-known for its anti-inflammatory effect via inhibition of 5-lipooxygenase expression. It has been proven that its anti-asthmatic effects could be boostered when patients use inhalative corticosteroids as a basic therapy. In a well-designed study, the extract of this herbal remedy was administered in form of oral tablets in patients with allergic asthma. In a six-months therapy, significant improvements in asthma signs and symptoms together with a reduced need of conventional asthmatic medications could be seen [31]. Similar results yielded with the use of *Sophora flavescens* in patients with refractory chronic asthma [32]. This herbal remedy proved to have anti-asthmatic characteristics in mast cell derived allergic inflammation diseases [33]. Purple passion fruit peel extract has been attributed to inhibit histamine release and involve in arachidonic acid metabolism. In a randomized, double-blinded study, effects of this herbal plant with dosage of 150 mg daily for four weeks treatment were compared to placebo; it has been shown that asthma symptoms like cough, wheezing, dyspnea, and force vital capacity (FVC) were significanty improved. However, forced expiratory volume in one second (FEV1) had no significant difference [34]. Two clinical trials studied the effects of combined formulas on asthmatic patients. In a crossover, randomized control study, effects of an oral treatment with a combined formula (*Picrorrhiza kurroa*, *apocynin*, *Picrorrhiza kurroa* and *Zingiber officinale*) and a standardized extract of *Ginkgo biloba* were investigated on therapy-refractory asthmatic patients. No significant improvements in asthma symptoms, pulmonary function test (PFT), and quality of life could be seen [35]. Another study with a herbal remedy (*Astragalus mongholius Bunge*, *Cordyceps sinensis Sacc.*, *Radix stemonae*, *Bulbus fritillariae cirrhosae,* and *Radix scutellariae*) was performed in pediatric asthma patients for six months with no improvement in lung function tests and other biometrical parameters [36].

### 3.4. Respiratory Tract Infection

Although upper respiratory tract infections are the most common infections in general population, lower respiratory tract infections are the leading cause of death among infectious diseases [37]. As phytomedicines have antibacterial, antiviral or immune modulatory effects, there are clinical evidences for the prevention and treatment of respiratory tract infections (Table 3).

*Pelargonium sidoides *extracts are widely used in the treatment of respiratory tract infections, e.g., acute rhinosinusitis and bronchitis. Its antimicrobial and anti-inflammatory characteristics are based on the release of tumor necrosis factor α (TNFα), nitric oxide, and increase in natural killer cells activity [38]. With the therapeutic dosage of 30 drops three times per day for at least seven days, patients show significant improvements in symptoms of common cold and acute bronchitis [23,39,40]. This is in accordance with a double-blind dose-finding study in which patients with acute bronchitis were treated with different dosages of *Pelargonium sidoides *extracts (tablet 10, 20, and 30 mg three times per day). The therapeutic results of these three treatment groups were superior to placebo with an optimal dose of 20 mg three times per day in consideration of the benefit risk ratio [41]. Thyme and primrose root are attributed with antibacterial, anti-fungal, and mucolytic characteristics. Patients treated with a combination of thyme and primrose root showed a significant reduction of cough, severity, and duration of acute bronchitis [42]. Ivy leaves extract represents another common medicinal plant used in the treatment of bronchitis due to its proven anti-inflammation, anti-congestion, antibacterial, and antispasmodic characteristics. A seven days oral treatment with ivy leaves extract reduced severity, and symptoms of acute or chronic bronchitis [43]. This accords with another study, in which two forms of ivy leaves extracts (Hedelix^®^ and Prospan^®^) significantly reduced severity of bronchitis compared to placebo [44]. Although adverse effects of ivy leaves extracts are low (2% of patients), it was indicated that the risk of occurance of side-effects could rise in concomitant therapy with antibiotics up to 26% of cases [43].

*Echinacea *has always been known to activate the immune system and anti-inflammatory reactions through the up-regulation of TNFα [45,46]. Therapeutic effects of *Echinacea *extract on sore throat and tonsillitis were investigated in two studies. In a double-dummy controlled trial, effects of *Echinacea *spray in combination with sage were compared to chlorohexidin/lidocain combination on sore throat. Interestingly, 60% of patients in each group became symptom-free after three days with no significant difference between these two combinations [47]. A Japanese herbal combination (*Gypsum*, *Radix bupleuri*, *Tuber pinelliae*, *Radix scutellariae*, *Radix platycodi*,* Fructus jujube*,* Radix panacis ginseng*, *Radix glycyrrhizae, *and *Rhizoma zingiberis*) was shown to be effective in chronic and recurrent tonillitis. After one year of treatment, the incidence of acute tonsillitis in patients with chronic tonsillitis decreased in seven of ten cases [48].

**Table 1:** Herbal remedies in the treatment of allergic rhinitis and acute rhinosinusitis.

Year	Author	Disease	Herbal remedy	Patient	Effects	Dosage
**2007**	**Yoshimura**	Allergic rhinitis	*naringenin chalcon*/tomato extracts	33	Inhibition of histamine release frommast cells	360 mg/oral/8 weeks
**2008**	**Corren**	Allergic rhinitis	*Cinnamomum zeylanicum, Malphighia glabra*, *Bidens pilosa*	20	Inhibition of neutrophil migration and mast cell production, reduction of histamine concentration	2 capsules/3 times per day/3 days
**2008**	**Tesche**	Acute viral rhinosinusitis	Cineole	150	Anti-inflammatory, accelerating the beat frequency of cilia and antimicrobial effects	200 mg/3 times per day/7 days
**2009**	**Bachert**	Acute rhinosinusitis	*Pelargonium sidoides*	103	Immune stimulant, inhibits bacterial adhesion to epithelial cells, stimulates cilliary beat frequency	60 drops/3 times per day/for maximum 22 days
**2009**	**Ciabatti**	Allergic rhinitis	*Capsicum oleous *	208	Activates/desensitizes sensory neurons or peptidergic nerve fibers	4 mg/puff, 3 times per day/3 days
**2009**	**Zhao**	Allergic rhinitis	Shi Bi Lin *	126	Suppression of eosinophil’s’ infiltration in the animals’ nasal tissues, decreasing of endothelial nitric oxide expression, inhibition of thromboxane B2 release	500 mg/2 times per day/14 days
**2010**	**Chui**	Allergic rhinitis	ARND **	35	Antibacterial , anti-inflammatory	2 sprays/3 times per day/7 weeks
**2010**	**Isik**	Allergic rhinitis	*Nigella sativa*	24	Increase in activity of phagocytosis and intracellular killing activity of PMNs, CD 8 counts, leukotriene synthesis and inhibition of histamine release	2 g oral per day/30 days
**2010**	**Matkovic**	Allergic rhinitis	*Astragalus membranaceus*	48	Decrease of INF-α, IL-5 and IL-13	80 mg capsules/1 per day/3 weeks
**2011**	**Nikakhlagh**	Allergic rhinitis	*Nigella sativa*	66	Increase of phagocytosis, intracellular killing activity of PMNs, CD 8 counts and leukotriene synthesis, inhibition of histamine release	2 g oral per day/30 days	
**2011**	**Yonekura**	Allergic rhinitis	*Rubus suavissimus (ten cha)*	87	Anti-inflammatory effects, inhibits releasing of histamine from mast cells	400 mg capsule/3 times per day/4 weeks

*: 7.5 g *Xanthium sibiricum* Patrin ex Widder *(Fructus asteraceae)*, 20 g *Angelica dahurica* (Fisch. ex Hoffm.) Benth. *(Radix apiaceae)*, 7.5 g *Saposhnikovia divaricata* (Turcz.) Schischk. *(Radix apiaceae)*, 15 g *Magnolia biondii* Pamp. *(Flos magnoliae)*, 5 g *Gentiana scabra* Bunge *(Radix gentianaceae)* and 5 g *Verbena officinalis* L. *(Herba verbenaceae)*. **: *Herba centipedae* 23%, *Herba menthae* 16%, *Radix paeoniae alba* 16%, *Radix scutellariae* 10%, *Radix glycyrrhizae* 6%, *Radix platcodi* 6%, *Flos lonicerae* 5%, *Fructus zizyphi jujubae* 5%, *Rhizoma coptidis* 4%, *Radix ledebouriellae* 5%, and *Pericarpium citri reticulatae* 4%.

**Table 2:** Herbal remedies in the treatment of asthma.

Year	Author	Herbal remedy	Patient	Effects	Dosage
**2007**	**Boskabady**	*Nigella sativa*	29	Relaxant, anticholinergic, antihistaminic	375 mL/kg 50% boiled extract for 2 months
**2007**	**Hoang**	*Sophora flavescens*	14	Anti-inflammatory activity, anti-asthmatic effect	4 g/2 times per day/3 months
**2007**	**Thomas**	AKL 1 *	32	Antibacterial, antioxidant, immunomodulatory, anti-inflammatory, anti-bronchospasm	500 mg/2 times per day/12 weeks
**2008**	**Watson**	purple passion fruit peel	-	Antioxidant, antiallergic, anti-inflammatory, decreasing of nitric oxide	150 mg/daily/4 weeks
**2009**	**Wong**	Combination of botanical product **	85	Antibacterial, antitussive, anti-inflammation	0.619 g capsule/daily/6 months
**2011**	**Belcaro**	French maritime pine bark extract (Pycnogenol)	76	Antioxidant, anti-inflammatory, vasodilatation	50 mg/2 times per day/6 months

*: *Picrorrhiza kurroa*, *apocynin*, *Zingiber officinale*, a standardized extract of *Ginkgo biloba*. **: *Astragalus mongholius Bunge*, *Cordyceps sinensis Sacc.*, *Radix stemonae*, *Bulbus fritillariae cirrhosae* and *Radix scutellariae*.

**Table 3:** Herbal remedies in the treatment of upper and lower respiratory tract infections.

Year	Author	Disease	Patient	Herbal remedy	Effects	Dosage
**2007**	Lizogub	Common cold	103	*Pelargonium sidoides *	Release of TNFα and nitric oxide, increasing the activities of natural killer cells	30 drops/3 times per day/10 days
**2007**	Kemmerich	Acute bronchitis	361	thyme herb/primrose root	Antibacterial, antifungal, mucolytic, antitussive	1 tab/3 times per day/11 days
**2007**	Schulz	Acute bronchitis	124	*Pelargonium sidoides*	Release of TNFα and nitric oxide, increasing the activities of natural killer cells	30 drops/3 times per day/7 days
**2007**	Matthys	Acute bronchitis	217	*Pelargonium sidoides*	Release of TNFα and nitric oxide, increasing the activities of natural killer cells	30 drops/3 times per day/7 days
**2007**	Kubo	Influenza	60	Mao-To	Antipyretic	0.06 g/kg body/3 times per day
**2008**	Vohra	Pediatric upper respiratory tract infections	46	Ginseng	Immunomodulatory, release of IL2, TNFα, IL 6, NO	600 mg and 400 mg/3 times per day/3 days
**2009**	Fazio	Acute and chronic bronchitis	9,657	Ivy leaf (*Hedera helix*)	Anti-inflammatory, anti-congestive, antibacterial, antispasmodic	3 times per day/7 days
**2009**	Schapowal	Sore throat		*Echinacea*/*Salvia*	*Echinacea*: anti-inflammatory, immune modulatory, upregulation of TNFα, antiviral and antibacterial, *Salvia*: anti-inflammatory, antibacterial, antinociceptive	2 puffs every 2 h
**2010**	Barrett	Common cold	713	*Echinacea*	Anti-inflammatory, immune modulatory, antiviral and antibacterial	1 tab/2 times per day/14 days
**2010**	Matthys	Acute bronchitis		*Pelargonium sidoides*	Release of TNFα and nitric oxide, increasing the activities of natural killer cells	tab 20 mg/1 or 2 or 3 times per day/7 days
**2010**	Goto	Chronic tonsillitis		Sho-saiko-to-ka-kikyo-sekko *	Not available	daily/12 months
**2010**	Kalus	Upper respiratory tract infections	300	*Cistus incanus* and Green Tea	Anti-inflammatory, antibacterial, antifungal and antispasmodic	12 tab/daily/7 days
**2010**	Sexana	Upper respiratory tract infections	223	*Andrographis paniculata*	Antibacterial, anti-inflammatory, immune stimulant, antipyretic	200 mg/day/5 days
**2010**	Wang	Influenza	480	Antiwei **	Antipyretic, antitussive	6g/BID/3 days
**2011**	Roll	Common cold	529	Juice powder concentrate of fruits and vegetables	increase in plasma levels of antioxidants and T-cells	4 capsules daily/8 months
**2011**	Cwientzeka	Acute bronchitis	590	Ivy leaf	Anti-inflammatory, anti-congestive, antibacterial, antispasmodic, sympatomimetic, secretion of surfactant	3 times per day/7 days

*: *Gypsum*, *Radix bupleuri*, *Tuber pinelliae*, *Radix scutellariae*, *Radix platycodi*, *Fructus jujube*, *Radix panacis ginseng*, *Radix glycyrrhizae* and *Rhizoma zingiberis*. **: Mahuang (*Herba Ephedra*), Baimaogeng (*Rhizoma Imperatae*), Gegen (*Radix puerariae*), Guizhi (*Ramulus Cinnamoumum*), Kuxingren (*Semen Armeniacae Amarum*.), Ganjiang (*Rhizoma Zingiberis*), Gancao (*Radix Glycyrrhizae*).

The therapeutic effect of *Echinacea* on acute viral respiratory infection (common cold) was studied with two species (*E. purpurea *and* E. angustifolia*). *Echinacea* tablets containing the equivalent of 675 mg *E. purpurea* root and 600 mg *E. angustifolia* root did not yield statistically significant shorter illness duration and lower severity. The authors trace this inefficiency back to the dose of the *Echinacea* formulation [49]. Preventive effects of a dietary supplement composed of a concentrated mixture of fruits and vegetables (Juice plus) on common cold were investigated in another study. This product contains antioxidants like vitamin B and C or β-carotene and is an immunomodulatory agent. Eight months administration of Juice plus could reduce the severity and duration of common cold [50]. *Camellia sinensis *has also a preventive role in flu and common cold in healthy patients. It can increase the interferon release and proliferation of T cells challenged *in vitro *[51].

A double dummy controlled trial compared the effects of *Mao-to* and oseltamivir as an antipyretic agent in Influenza typ A. This remedy is known as an immune modulator and stimulates the release of interferon β from host cells. In this study, patients were categorized in three groups (oseltamivir alone, *Mao-to *alone and combined therapy of these two). *Mao-to* powder alone or as a combination with oseltamivir tablets could reduce fever in patients significantly [52]. A three-day treatment with Antiwei tablets, a herbal mixture (see Table 3), has indicated to be significantly effective on severity and duration of Influenza in comparison to placebo. This mixture in tablet form has antipyretic and antitussive effects [53].

Green tea has antibacterial, antitoxic, antiviral, and antifungal activities in upper and lower respiratory tract infections [54]. In the same manner, *Cistus incanus *extract (CYSTUS052) has also shown antibacterial, antiviral, anti-inflammatory, and antispasmodic effects in different diseases. *Cistus incanus* has been indicated to have a better effect on duration and severity in viral and bacterial infections of the upper respiratory tract compared to green tea [55]. Ginseng stimulates the immune system and enhances the production of interleukin 2, TNFα, interlukeukin 6, nitric oxide in murine spleen cells [56]. In a control trial study in order to determine safety and dosage of ginseng, children with upper respiratory tract infections were treated in three arms with different ginseng dosages. The standard dose (600 mg TID day 1, 400 mg TID day 2 and 200 mg TID day 3) and half of the standard dose of ginseng were compared to a placebo group. Frequency and severity of respiratory tract infections and reported adverse events were not significantly different among these three arms [57]. In contrast, *Andrographis paniculata* tablets (200 mg/day) in a 5-day treatment were superior to placebo in the treatment of upper respiratory tract infection syndroms [58].

### 3.5. Chronic Obstructive Pulmonary Disease (COPD)

Chronic inflammation, related respiratory tract stenosis and impaired mucocilliary movements in chronic obstructive pulmonary disease (COPD) lead to dyspnea, chronic cough with sputum and impaired pulmonary function tests. COPD patients can be treated with antibiotics, bronchodilators, and corticosteroids [59].

*Hochuekkito*, containing ten Japanese herbs, has been known to have anti-inflammatory effects. In a six-months study, with the dosage of 2.5 mg tablets three times per day plus bronchodilators as a basic treatment in elderly patients with COPD, patients had better FEV1, TNFα and c-reactive protein blood and interleukin 6 level in comparison to the placebo group [60]. *Echinacea purpurea* (EP) is known as an immune modulator and can help in induction of apoptosis, cell migration and inhibition of TNFα. The effect of EP, placebo and EP plus micro-nutritions (zinc, selenium and Vitamin C) were compared with each other for seven days [61]. Since only the EP+ group had better results than placebo, it seems that in a short time, the effect of this herbal remedy could be boosted in accompanying with micro-nutritions [62].

Since *cineole* has positive effects on the beat frequency of the cilia and bronchodilation, it may show positive influence on the exacerbations of COPD and lung function tests [27,63]. Patients who received 200 mg of *cineole* tablets three times a day had a reduction of exacerbations such as dyspnea and improvements of lung function and health status [64].

### 3.6. Otitis Media

Recurrent otitis media in young children has been tried to treat with *Echinacea* in combination with osteopathic manipulative treatment (OMT). For this purpose, an American group of pediatricians conducted a randomized, placebo-controlled trial with a six-month follow-up over four years. Children aged 12–60 months with recurrent otitis media received an alcohol extract of *Echinacea purpurea* roots and seeds (or placebo) for ten days at the first sign of common cold. Five OMT visits (or sham treatments) were offered over three months. Remarkably, *Echinacea purpurea* was associated with a borderline increased risk of having at least one episode of acute otitis media during six-months follow-up compared to placebo, whereas no interaction was found between *Echinacea* and OMT [65]. 

### 3.7. Ideopatic Hearing Loss and Tinnitus

Ideopathic sudden hearing loss and tinnitus aurium represent diseases frequently treated in Otorhinolaryngology. Pentoxifylline and cortisone represent long-established drugs against sudden hearing loss and acute tinnitus. Although there is a lack of definitive evidence-based data, they are still in clinical use. This dillema promotes the trying of complementary and alternative medicine. *Ginkgo biloba* represents one of the most used phytomedicines in Otorhinolaryngology against sudden hearing loss and tinnitus. The efficacy of ginkgo special extract EGb 761 in patients with chronic tinnitus was studied. In this trial, patients received a 10 days intravenous treatment with 200 mg/day EGb 761, after they were randomized to a double-blind oral treatment with either 2 × 80 mg/day EGb 761 or placebo with a treatment period of 12 weeks. Interestingly, the combination of intravenous followed by oral treatment with ginkgo special extract EGb 761 appeared to be effective and safe in reducing the individual sound intensity of tinnitus [66]. In another trial, EGb 761 was compared to a conventional treatment with pentoxifylline for sudden hearing loss. Treatment with EGb 761 was as effective as treatment with pentoxifylline [67]. In a multicentre, randomized, double-blind phase III study, administered oral doses of 120 mg twice daily and 12 mg twice daily over eight weeks were compared. It is reported that the higher dose of EGb 761 appears to accelerate the recovery of patients with sudden hearing loss less than 75 dB, with a good chance of complete recovery [68].

A treatment combination of *aescin *and* troxerutin* against hearing loss caused an improvement in the hearing level (>10 dB) of patients in contrast to pentoxyfylline [69]. It has shown that *aescin* can induce endothelial nitric oxide synthesis and is an angatonist of serotonin and histamine. These effects can help in treating inflammation, fluid collection, and thereby improve the sound conduction process [70].

### 3.8. Oral Cavity

The *Eucalyptus globulus* leave is reported to have antibacterial activity against oral bacteria, such as *Streptococcus mutans* [71]. *Eucalyptus* extracts in chewing gum has been shown to significantly inhibit plaque formation, inflammation, and bleeding of gingiva [72]. *Streptococcus mutans* activity can also be inhibited by *Terminalia chebula* as it increases the pH-value of saliva [73].

Leaves of myrtle (*Myrtus communis*) have been attributed with antibacterial, analgesic, anti-inflammatory, and free-radical scavenging activities. In a controlled trial, the application of a paste containing 5% myrtle was effective in the treatment of recurrent aphtus stomatitis. It could significantly reduce ulcer size, pain severity, erythema, and exudation level of recurrent aphthous stomatitis [74]. *Rosa damascene* is known to have antibacterial, antioxidant, antitussive, hypnotic, and antidiabetic effects. The results of one study showed that a mouthrinse containing *Rosa damascena* extract was more effective although not more significant than the placebo in severity of pain, size, and number of the ulcers of recurrent aphthous stomatitis [75].

### 3.9. Oncology

Chinese herbal recipe, “Feiji Recipe” was reported to improve clinical symptoms of middle stage and advanced non-small cell lung cancer (NSCLC). The remedy decoction consists of: *Radix astragali *30 g, *Radix glehniae* 15 g, *Tuber ophiopogonis japonici* 12 g, *Tuber asparagi cochinchinenses* 12 g, *Sclerotium poriae cocos* 15 g, *Selaginelladoed* 30 g, *Salvia chinensia* 30 g, *Herba cum radice houttuyniae cordatae* 30 g and *Houttuynia cordata* 24 g. In a randomized, controlled pilot study, simultaneous application of “Feiji Recipe” and chemotherapy in patients with NSCLC could prevent the worsening of function in terms of role, social, fatigue, and global health evaluated with the Core Quality of Life Questionnaire (QLQ-C30) [76].

Green tea is attributed to various healthy benefits, e.g., prevention of cardiovascular diseases and cancer in preclinical studies [77]. From the M.D. Anderson Cancer Center in Houston comes a phase II randomized, placebo-controlled trial with green tea extract (GTE) in patients with high-risk oral premalignant lesions. Patients receiving high doses of green tea extract GTE (750 and 1,000 mg/m2) three times daily for 12 weeks showed clinical response and improvements of histology [78]. GTE contains high amounts of polyphenols, including epigallocatechin 3-gallate (EGCG) and could prevent oral cancer in preclinical models [79]. EGCG regulates apoptosis and blocks angiogenesis by decreasing phosphorylation of vascular endothelial growth factor receptor and inhibits its release from tumor cells [80].

Rhubarb has been well-known in Chinese medicine for its medical properties*. *In a recent study, rhubarb extract significantly attenuated radiation, induced lung toxicity, and improved pulmonary function. This prophylactic effect of rhubarb on lung cancer patients treated with radiotherapy has been explained by decreasing the level of transforming growth factor-beta-1 and interleukin-6 [81].

### 3.10. Post-Operative Management

Herbal remedies can change the pain score in post-operative patients. *Arnica montana* has been found to have analgesic effects [82]. *Arnica montana* extract administered to patients after tonsillectomy significantly decreased their pain scores compared to the placebo group. However, significant changes in analgesic consumption were not noted [83]. In another post-operative study*, Melilotus *extract has been used in the treatment of ecchymosis after rhinoplasty and blepharoplasty. It could decrease ecchymosis and paranasal, upper, and lower eyelids edema compared with the control group [84].

## 4. Discussion

Phytomedicine has become an important alternative treatment option in the western world [8,85]. Huge financial, natural and human resources have been spent in this field. The question arises whether this development is justified and can be objectively proven. More than ever before, western physicians demand clinical evidence for the efficacy of phytomedicines in appropriately designed clinical trials. In overview of all publications, there is a need in clarifying whether phytomedical agents could substitute a classical therapy considering efficiency and side-effects. In addition, it would be reasonable to compare effects of herbal remedies with placebo and double dummies. We found four studies with a double dummy design [10,47,52,69]. Furthermore, there is a lack of dose-finding studies. In fact, only two studies evaluated the dosage of phytotherapeutics in patients over the last five years [10,41]. 

Measurement tools are a very important concern in evaluating the efficiency of therapeutic means. Among the studies reviewed, 27.2% were evaluated subjectively, e.g., quality of life questionnaires or visual analogue scales. Only 16.3% of studies were evaluated with objective measurement tools, e.g., cell counts and function tests. Interestingly, a significant percentage of studies (56.3%) applied both, subjective and objective measurement tools. 

Remedies in studies were applied as single or combinations of several phytotherapeutics, e.g., in the form of decoctions. In the latter case, it is difficult to determine the degree of therapeutical contribution of each component. Pharmacological effects are considered to be the result of the synergistic or antagonistic interaction of many phytochemicals [7]. However, it has been shown that a single herbal compound can have a less pharmacological effect than a combination of compounds [28]. In this review, 20% of study results were non-significant with combinations of medicinal plants *versus* 10% with single herbs.

Evidently, there are several weak and recurrent aspects in the majority of publications in PubMed. Among these are short duration of treatment periods, small sample sizes, inappropriate randomization, blinding, and controls. Application of unknown effective dosages, ignorance of underlying diseases, and patients’ age may also have negative influence on therapeutical efficacy, and statistical power [86]. Moreover, we experienced in our Medline search that a critical number of publications from China are not written in English and/or are not provided with abstracts. Considering the time period from January 2007 until April 2012 with our inclusion criteria and with all available languages, Medline search yielded 1,183 articles covering 322 non-English written publications. Among those, 280 articles were published in Chinese language. Although titles of studies promise to be of clinical interest, further information, and results of individual trials are not provided except for the Chinese-speaking peer-reviewing community. This represents one of various major obstacles in achieving recognition of phytomedicine in western science. Clinical and scientific development can only be assured if experiences are worldwide published in English.

Depending on the chemical contents of each herb, the method of extraction, and also the season of its harvest, efficiency reports of herbal remedies are sometimes inconsistent [57,87]. Another neglected aspect for controversial results in clinical trials is the ignorance of quality control. Quality control comprises the correct identification and authentication of medicinal plants. Moreover, quality control ensures not only proper composition of herbal remedies but also prevents contamination of herbal drugs with mycotoxins, pesticides, heavy metals, and chemical toxins with advanced techniques, e.g., high-performance liquid chromatography fingerprints and random amplified polymorphic DNA analysis [6]. Qualitive and quantitive heterogeneity of production might be a restrictive factor in the clinical evaluation of herbal remedies [88] and represent an important factor of safety for patients. Reports of toxicity vary on available medical guidelines and recommended doses in each country [57,89]. A stepwise evaluation of phytomedical products in phase I to phase III trials, as it is routinely done in pharmaceutical research, would help to clarify this issue.

It must be emphasized that efficacy and safety of phytotherapeutics depend on the quality of phytomedical products. Appropriate quality assurance and control of herbal drugs as well as sustainable production methods are pre-conditions for the implementation of phytomedicine in cancer therapy at an international level [90]. Another neglected aspect for controversial results in clinical trials is the ignorance of quality control. Quality control comprises the correct identification and authentication of medicinal plants. Moreover, quality control ensures not only proper composition of herbal remedies but also prevents contamination of herbal drugs with mycotoxins, pesticides, heavy metals, and chemical toxins with advanced techniques, e.g., high-performance liquid chromatography fingerprints and random amplified polymorphic DNA analysis [6].

## 5. Conclusions

Phytomedicine is founded on millenia-old knowledge of medicinal plants. Today, it has become a segment of the Asian and even European pharmaceutical market. We have existing and convincing evidence-based clinical data in international databases, e.g., PubMed (Medline). However, recognition and application of phytomedicine into western medicine will depend on credible evidence-based clinical data. Phytomedicine will only enter in professional clinical application if safety, efficacy and quality are proven in a comparable manner to conventional drugs. For this purpose, it is mandatory to conduct well-designed clinical trials. Awareness of Good Clinical Trial Practice and provision of knowledge worldwide is the existential foundation for proper scientific development.
